# Loss of tricellular tight junction tricellulin leads to hyposalivation in Sjögren’s syndrome

**DOI:** 10.1038/s41368-025-00349-9

**Published:** 2025-03-19

**Authors:** Xiangdi Mao, Haibing Li, Sainan Min, Jiazeng Su, Pan Wei, Yan Zhang, Qihua He, Liling Wu, Guangyan Yu, Xin Cong

**Affiliations:** 1 Department of Physiology and Pathophysiology, Peking University School of Basic Medical Sciences, State Key Laboratory of Vascular Homeostasis and Remodeling, Beijing, China; 2 Department of Oral and Maxillofacial Surgery, Peking University School and Hospital of Stomatology & National Center of Stomatology & National Clinical Research Center for Oral Diseases & National Engineering Research Center of Oral Biomaterials and Digital Medical Devices, Beijing, China; 3 Department of Oral Medicine, Peking University School and Hospital of Stomatology & National Center of Stomatology & National Clinical Research Center for Oral Diseases & National Engineering Research Center of Oral Biomaterials and Digital Medical Devices, Beijing, China; 4 State Key Laboratory of Natural and Biomimetic Drugs, Peking University, Beijing, China

**Keywords:** Mechanisms of disease, Salivary gland diseases

## Abstract

Tricellulin, a key tricellular tight junction (TJ) protein, is essential for maintaining the barrier integrity of acinar epithelia against macromolecular passage in salivary glands. This study aims to explore the role and regulatory mechanism of tricellulin in the development of salivary gland hypofunction in Sjögren’s syndrome (SS). Employing a multifaceted approach involving patient biopsies, non-obese diabetic (NOD) mice as a SS model, salivary gland acinar cell-specific tricellulin conditional knockout (*Tric*^*CKO*^) mice, and IFN-γ-stimulated salivary gland epithelial cells, we investigated the role of tricellulin in SS-related hyposalivation. Our data revealed diminished levels of tricellulin in salivary glands of SS patients. Similarly, NOD mice displayed a reduction in tricellulin expression from the onset of the disease, concomitant with hyposecretion and an increase in salivary albumin content. Consistent with these findings, *Tric*^*CKO*^ mice exhibited both hyposecretion and leakage of macromolecular tracers when compared to control animals. Mechanistically, the JAK/STAT1/miR-145 axis was identified as mediating the IFN-γ-induced downregulation of tricellulin. Treatment with AT1001, a TJ sealer, ameliorated epithelial barrier dysfunction, restored tricellulin expression, and consequently alleviated hyposalivation in NOD mice. Importantly, treatment with miR-145 antagomir to specifically recover the expression of tricellulin in NOD mice significantly alleviated hyposalivation and macromolecular leakage. Collectively, we identified that tricellulin deficiency in salivary glands contributed to hyposalivation in SS. Our findings highlight tricellulin as a potential therapeutic target for hyposecretion, particularly in the context of reinforcing epithelial barrier function through preventing leakage of macromolecules in salivary glands.

## Introduction

Material transport across epithelial cells in exocrine glands occurs via two primary routes: the transcellular pathway, mediated by transporters, and the paracellular pathway, governed by tight junctions (TJs).^1,2^ TJs are localized at the apicolateral membranes of adjacent polarized cells and consist of transmembrane proteins, including claudins, occludin, and junctional adhesion molecules (JAMs), as well as cytoplasmic proteins, such as ZO proteins and cingulin.^3,4^ Over the past two decades, significant research efforts have been directed toward the identification of tricellular TJs (tTJs). Tricellulin, encoded by *MARVELD2* (also known as *TRIC*) gene, was the first tTJ protein to be identified.^5^ At the tripartite cell junction, tricellulin constructs a ~10 nm “central tube” through a vertically orientated triple-pair strand structure, thereby forming a barrier that predominantly restricts the passage of macromolecules—a function distinct from that of bicellular TJs (bTJs), which regulates ion permeability.^6^ It is widely acknowledged that the abnormal expression of tricellulin or the presence of mutants can lead to alterations in the epithelium barrier. In tricellulin-knockout EpH4 cells, the tTJ integrity is interrupted, leading to a porous and dysfunctional barrier incapable of effectively preventing the passage of macromolecules.^7^ In MDCK cells, the overexpression of tricellulin decreases the paracellular permeability of macromolecules (*Mr* > 4 kD).^8^ During the early stages of ulcerative colitis, the IL-13-induced downregulation of tricellulin results in an increase in macromolecule permeability across the colon epithelium.^9^ Consistent with these findings, our previous studies documented an increased presence of macromolecules in the saliva of immunoglobulin-like domain-containing receptor 1^−/−^ (ILDR^−/−^) mice, accompanied by the redistribution of tricellulin in salivary glands.^10^ These observations further revealed that suppression of tricellulin leads to a heightened permeability of 40 kDa fluorescein isothiocyanate-dextran (FD40) across the salivary gland epithelium.^10^ Nonetheless, the exact role of tricellulin in saliva secretion remains to be fully elucidated.

Sjögren’s syndrome (SS), a systemic autoimmune disorder, is characterized by lymphocytic infiltration and diminished secretory function in exocrine glands, prominently affecting salivary and lacrimal glands. The clinical manifestations of xerostomia (dry mouth) and xerophthalmia (dry eyes) in SS patients considerably compromise their quality of life.^11–13^ The prevailing hypothesis posits that dysregulated innate and adaptive immunity, culminating in an escalation of pro-inflammatory cytokines, is pivotal to the initiation and progression of SS.^14–16^ As a result, therapeutic strategies targeting the modulation of the dysfunctional immune system have emerged as effective treatments for SS.^17,18^ Nevertheless, recent investigations suggest that the correlation between lymphocytic infiltration and salivary gland dysfunction in SS patients is less pronounced than previously presumed.^19,20^ This observation underscores the importance of exploring therapies that enhance glandular epithelial function in the context of SS management. Moreover, the disruption of the epithelial barrier in salivary glands has been implicated as a critical factor in SS pathogenesis. In labial salivary glands (LSGs) obtained from SS patients, the expression patterns and distributions of various TJ proteins, including ZO-1, occludin, and claudin-1, -3, and -4, are altered by focal pro-inflammatory cytokines.^21^ Similarly, in the submandibular glands (SMGs) of non-obese diabetic (NOD) mice, a recognized animal model for SS, there is an observed increase in the clearance of paracellular tracers, a widened acinar TJ zone, and aberrant expression profiles of multiple TJ proteins. These changes are attributed to the infiltration of Th17 lymphocytes.^21,22^ However, the precise mechanisms by which the epithelial TJ barrier contributes to SS progression require further investigation. Furthermore, given the marked elevation of macromolecules, such as autoantibodies, inflammatory factors, and other proteins, in the saliva of SS patients,^23,24^ the potential role of tricellulin in mediating this pathological secretory pattern remains unexplored. This knowledge gap highlights the necessity for additional research to elucidate the specific contribution of tricellulin to the disrupted salivary secretion in SS.

To investigate the expression profile of tricellulin in salivary glands during SS, we analyzed transcriptomic datasets derived from SS patients and healthy controls. Our analysis revealed a consistent downregulation of tricellulin in SS, which we corroborated through immunohistochemical examination of salivary gland tissue samples from both SS patients and NOD mice. By establishing a previously unrecognized salivary gland acinar cell-specific tricellulin conditional knockout mouse model, we assessed the implications of tricellulin deficiency on macromolecule transport across the salivary gland epithelium. Furthermore, we probed the dynamics of tricellulin expression under inflammatory conditions of SS in both mouse models and cell cultures, aiming to unravel the complex interplay between inflammation and tricellulin regulation.

## Results

### Expression of tricellulin is reduced in salivary glands of SS patients

**Fig. 1:**
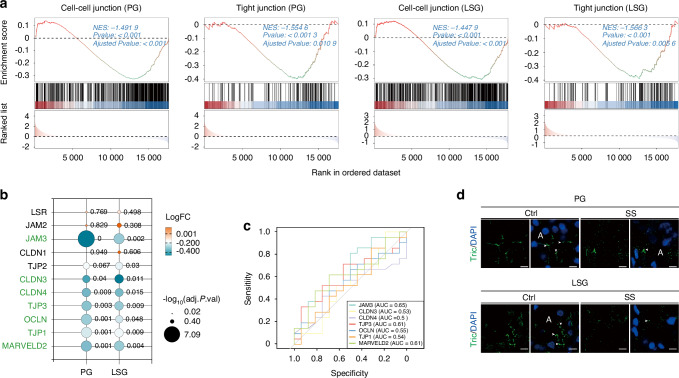
The expression of tricellulin in salivary gland biopsies from Sjögren’s syndrome (SS) patients. **a** Gene Set Enrichment Analysis (GSEA) results showing cell junction-related pathways. PG parotid gland. LSG labial salivary gland. **b** The differential expressions of tight junction (TJ)-related genes. The green color-labeled gene names were presented as the downregulated TJs. The number presented was the significant value. LSR lipolysis-stimulated lipoprotein receptor, JAM junctional adhesion molecule, CLDN claudin, TJP tight junction protein, OCLN occludin, MARVELD2 membrane-associating domain containing 2, Tric tricellulin. **c** The receiver operating characteristic curve (ROC) showing the diagnostic effects of TJ mRNA expression levels in parotid glands from SS patients. **d** The distribution of tricellulin in salivary glands of control and SS patients. Arrows pointed to the localization of tricellulin. Cell nuclei were stained with DAPI (blue). Bar: 8 µm. *n* = 1 for PG biopsy and *n* = 6 for LSG biopsies. Ctrl control, A acini

To explore the role of tricellulin and other TJs in SS, we analyzed RNA sequencing datasets (GSE173808 and GSE208260). Patient selection adhered to the 2016 ACR-EULAR criteria and histopathological phenotyping. The biopsy-negative non-SS sicca subjects served as the control group, while the biopsy-positive SS patients served as the SS group. The analysis encompassed 37 parotid gland (PG) samples (16 controls, 21 patients) and 50 LSG samples (17 controls, 33 patients). Gene Set Enrichment Analysis (GSEA) showed diminished activity in cell-cell junction-related pathways and TJ expression in both PGs and LSGs from SS patients compare to controls (Fig. 1a). Specifically, the mRNA levels of tricellulin (*MARVELD2*), ZO-1 (*TJP1*), occludin (*OCLN*), ZO-3 (*TJP3*), claudin-3 (*CLDN3*), claudin-4 (*CLDN4*), and junctional adhesion molecule 3 (*JAM3*) were markedly lower in PGs and LSGs from SS patients (Fig. 1b). The receiver operating characteristic (ROC) curve analysis demonstrated that tricellulin, ZO-3, and JAM3 mRNA expression in PGs had a certain diagnostic accuracy for SS (Fig. 1c). We then assessed tricellulin expression patterns in SS biopsy specimens. Histological staining highlighted obvious lymphocytic infiltration in PGs and LSGs of SS patients (Supplemental Fig. 1). Immunofluorescence images revealed that tricellulin was predominantly localized at the apicolateral membranes between adjacent acini in control salivary glands, with a notable reduction in intensity in SS samples (Fig. 1d). These data collectively indicate a downregulation of tricellulin in salivary glands of SS.

### Expression of tricellulin is decreased in hypofunctional SMGs of NOD mice

**Fig. 2:**
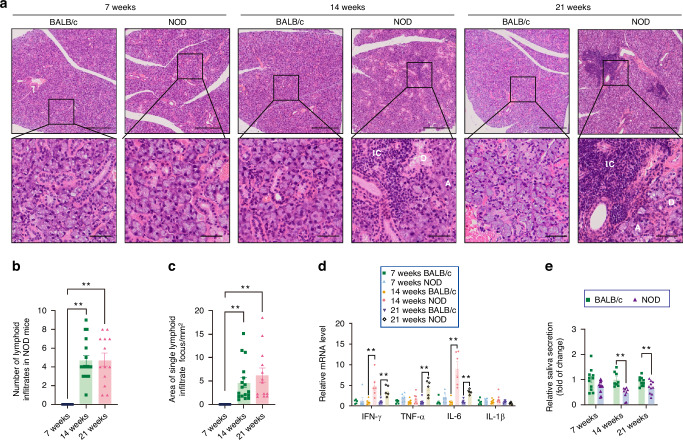
The occurrence of spontaneous salivary gland inflammation in non-obese diabetic (NOD). **a** Hematoxylin and eosin (H&E) staining of SMGs. The enlarged images (bar: 50 µm) were derived from boxes in the upper panels (bar: 250 µm). IC infiltrating cells, A acini, D duct. **b**, **c** The number of lymphoid infiltrates (**b**) and area of single infiltrates focus (**c**) in SMGs of NOD mice. *n* = 9–18. **d** The mRNA expressions of inflammatory factors in SMGs. The mRNA levels in NOD mice were presented as the relative ratio to that in BALB/c mice. *n* = 5–6. IFN-γ interferon-γ, TNF-α tumor necrosis factor-α, IL-6 interleukin-6, IL-1β interleukin-1β. **e** The stimulated saliva secretion in mice. The saliva secretion in NOD mice was presented as the relative ratio to that in BALB/c mice. *n* = 7–12. Analysis was performed by using Kruskal-Wallis’ test (**b**, **c**) and unpaired two-tailed *t* test (**d**, **e**) where *P < 0.05 and **P < 0.01. The data are presented as means ± SEM (**d**, **e**)

**Fig. 3:**
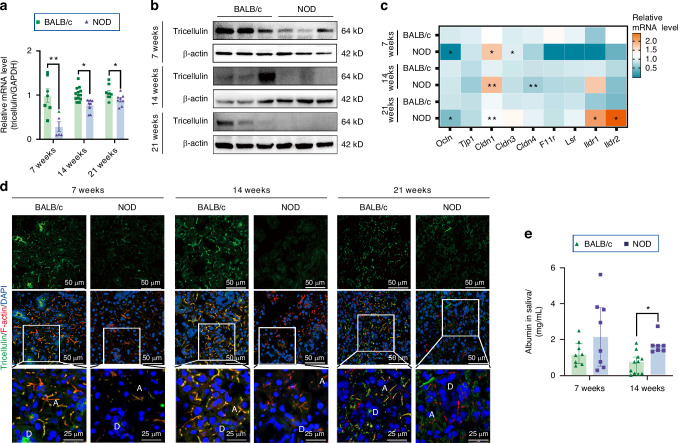
The expression of tricellulin in submandibular glands (SMGs) of non-obese diabetic (NOD) mice. **a**, **b** The mRNA (**a**) and protein (**b**) expression of tricellulin in SMGs. The expression of tricellulin in NOD mice was presented as the relative ratio to that in BALB/c mice. *n* = 5–13. **c** The mRNA expressions of tight junction proteins in SMGs. Ocln occludin, Tjp1 tight junction protein 1, Cldn claudin, F11r junctional adhesion molecule 1, Lsr lipolysis-stimulated lipoprotein receptor, Ildr immunoglobulin-like domain containing receptor. **d** The co-immunostaining of tricellulin (green) and F-actin (red). The enlarged images (bar: 25 µm) were derived from boxes in the upper panels (bar: 50 µm). Cell nuclei were stained with DAPI (blue). **e** The level of albumin in saliva. *n* = 7–11. Analysis was performed by using unpaired two-tailed *t* test (**a**, **c**) and Mann-Whitney’s test (**e**) where *P < 0.05 and **P < 0.01. The data are presented as means ± SEM (**a**, **e**) or means (**c**)

Next, to elucidate alterations in tricellulin during the onset and progression of SS, we utilized differently aged NOD mice, a well-established animal model that recapitulates features of Sjögren’s-like syndrome.^25,26^ At 7 weeks of age, no discernible histological differences were apparent in the SMGs of NOD mice relative to BALB/c mice. However, at 14 and 21 weeks, there was a significant increase in both the quantity and size of lymphocytic foci in NOD mice compared to age-matched BALB/c mice (Fig. 2a–c). Additionally, we have performed the immunofluorescence staining of CD3 and CD4 for T cells and CD4^+^ T cells (Th) cells, respectively. Results showed an obvious infiltration of CD3^+^ and CD4^+^ T cells in the SMGs of 14-week-old NOD mice compared to controls, and this infiltration became severer in the SMGs of 21-week-old NOD mice (Supplemental Fig. 2a). We also analyzed the correlation between T cell infiltration and tricellulin expression according to public datasets (GSE173808 and GSE208260). There was a negative correlation between tricellulin gene expression and those of CD3D, CD3E (two important CD3 subunits), and CD4, with Pearson correlation coefficients of −0.58, −0.55, and −0.46 respectively (Supplemental Fig. 2b), suggesting that the quantity of T cells may negatively correlate with tricellulin expression in the salivary glands of patients with SS. Additionally, the mRNA expression of proinflammatory cytokines, specifically interferon-γ (IFN-γ) and IL-6, was elevated in SMGs of 14- and 21-week-old NOD mice, whereas the upregulation of tumor necrosis factor-α (TNF-α) mRNA was exclusive to 21-week-old NOD mice. Interestingly, the mRNA level of IL-1β remained unchanged between NOD and BALB/c mice (Fig. 2d). The salivary flow rate was significantly decreased in both 14- and 21-week-old NOD mice, though not in 7-week-old NOD mice (Fig. 2e). Notably, tricellulin mRNA and protein expression were markedly reduced in the SMGs of NOD mice across all three age groups (7, 14, and 21 weeks) when compared to age-matched BALB/c mice (Fig. 3a, b, Supplemental Fig. 2c). Moreover, qPCR analysis revealed dysregulated expression of TJ components. We observed reduced occludin and elevated claudin-1 and claudin-3 mRNA in 7-week-old NOD mice, increased claudin-1 and decreased claudin-4 mRNA in 14-week-old NOD mice, and decreased occludin alongside increased claudin-1, immunoglobulin-like domain containing receptor 1 (ILDR1), and ILDR2 mRNA in 21-week-old NOD mice, all compared to their respective age-matched BALB/c mice (Fig. 3c). Immunofluorescence images further revealed a decline in tricellulin intensity at the apicolateral membranes of acinar cells in 14- and 21-week-old NOD mice, with a trend towards reduced tricellulin apparent even at 7 weeks of age (Fig. 3d, Supplemental Fig. 2d).

To evaluate the potential implications of tricellulin absence in acinar epithelial cells in NOD mice, we quantified the concentration of albumin (a prototypical macromolecule that traverses via the paracellular route) in saliva. Compared to age-matched BALB/c controls, the salivary albumin content displayed a rising trend in 7-week-old NOD mice and a statistically significant elevation in 14-week-old NOD mice (Fig. 3e). These observations indicate that the downregulation of tricellulin expression is concurrent with both hyposecretion and enhanced permeability to macromolecules, phenomena that escalate as the disease progresses.

### Deficiency of tricellulin leads to hyposalivation and leakage of macromolecules

**Fig. 4:**
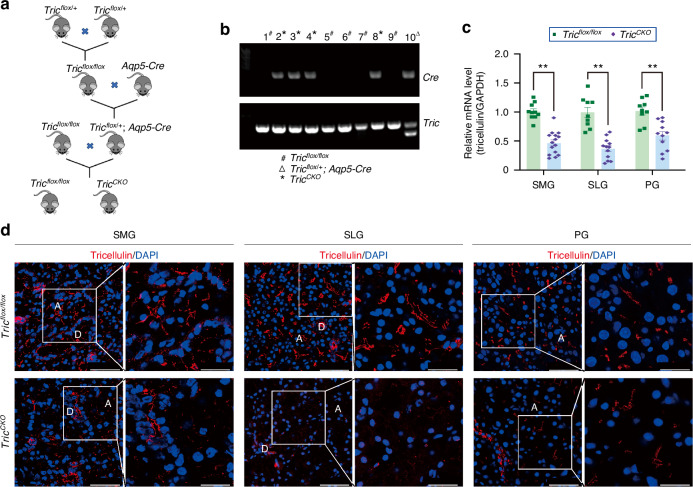
The establishment of salivary gland acinar cell-specific tricellulin conditional knockout (*Tric*^*CKO*^) mice. **a** The breeding process of *Tric*^*CKO*^ mice. **b** Genotype identification of *Tric*^*CKO*^ mice. Number 2–4 and 8 mice were *Tric*^*CKO*^ mice, number 5–7 and 9 mice were *Tric*^*flox/flox*^ mice, and number 10 mouse was *Tric*^*flox/+*^ mice. **c** The mRNA level of tricellulin in salivary glands. The mRNA levels in *Tric*^*CKO*^ mice were presented as the relative ratio to that in *Tric*^*flox/flox*^ mice. *n* = 9–15. SMG, submandibular gland. SLG sublingual gland, PG parotid gland. **d** The distribution of tricellulin (red) in salivary glands. Cell nuclei were stained with DAPI (blue). The enlarged images (bar: 25 µm) were derived from boxes in the left panels (bar: 50 µm). Analysis was performed by using unpaired two-tailed *t* test (**c**). The data are presented as means ± SEM (**c**)

To ascertain whether there exists a causal relationship between tricellulin absence and abnormal secretory patterns, we engineered salivary gland acinar cell-specific tricellulin conditional knockout mice. This was achieved by selectively ablating the *Tric* gene in salivary gland acini through the Cre-LoxP recombination system. Genotyping of tail DNA confirmed the successful generation of *Tric*^*CKO*^ mice (Fig. 4a, b). Notably, the mRNA expression of tricellulin was markedly reduced in major salivary glands, including SMGs, SLGs, and PGs (Fig. 4c). Moreover, the mRNA level of tricellulin was also significantly diminished in the lung tissue (Supplemental Fig. 3a), given that AQP5, a marker used for Cre expression, is also expressed in the lung.^27^ Immunofluorescence analysis further corroborated these findings, revealing a substantial decrease in tricellulin signals in the acinar regions of salivary glands (Fig. 4d, Supplemental Fig. 3b). These results confirm the establishment of a functional acinar cell-specific tricellulin conditional knockout mouse model.

**Fig. 5:**
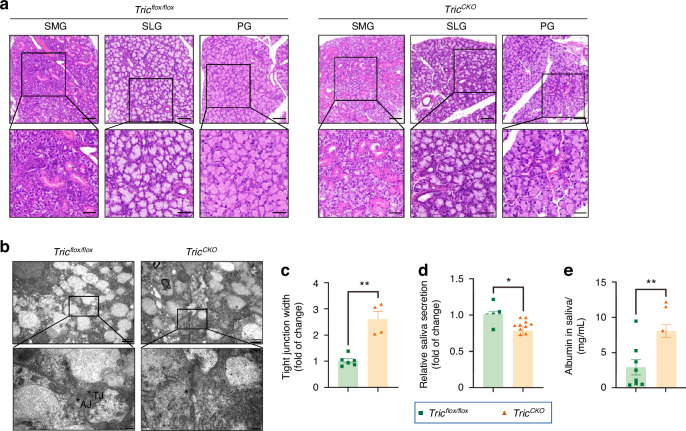
The barrier function of tight junction in salivary gland acinar cell-specific tricellulin conditional knockout (*Tric*^*CKO*^) mice. **a** Hematoxylin and eosin (H&E) staining of salivary glands. The enlarged images (bar: 50 µm) were derived from boxes in the upper panels (bar: 100 µm). SMG submandibular gland. SLG sublingual gland, PG parotid gland. **b** The ultrastructures of cell junctions by transmission electron microscope. The enlarged images (bar: 200 nm) were derived from boxes in the upper panels (bar: 1 µm). Arrows pointed to tight junction (TJ) and asterisk pointed to adherens junction (AJ). **c** The width of tight junction in acini of SMGs. The width was calculated by the Image J software according to the enlarged images (**b** bar: 200 nm). *n* = 4–5. **d**, **e** The saliva secretion (**d**) and the albumin level in saliva (**e**) in *Tric*^*CKO*^ mice. *n* = 7–12. Analysis was performed by using unpaired two-tailed *t* test (**c**–**e**) where *P < 0.05 and **P < 0.01. The data are presented as means ± SEM (**c**–**e**)

**Fig. 6:**
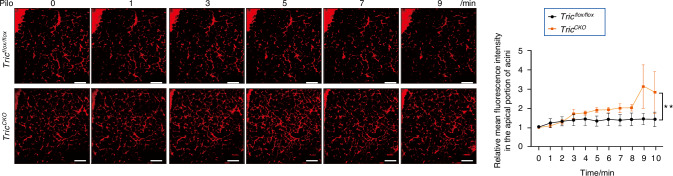
An in vivo permeability assay was performed by injecting 40 kDa rhodamine B-labeled dextran (RD40) into the angular vein of mice. The unilateral submandibular gland (SMG) was isolated and placed on a glass chamber under a two-photon laser-scanning microscope. For the semi-qualification analysis, the fluorescence of RD40 within the lumen and around the basal sides of acini was measured by the Image J software. *n* = 3. Pilo pilocarpine. Analysis was performed by using 2-way ANOVA where *P < 0.05 and **P < 0.01. The data are presented as means ± SEM. bar = 100 μm

In *Tric*^*CKO*^ mice, the histological examination showed no apparent alterations in the architecture of either the salivary glands or lungs, indicating that the overall tissue structure remained intact (Fig. 5a, Supplemental Fig. 3c, d). Nevertheless, a striking expansion of TJ width between neighboring acinar cells was evident in *Tric*^*CKO*^ mice compared to *Tric*^*flox/flox*^ controls (Fig. 5b, c). Remarkably, *Tric*^*CKO*^ mice manifested reduced saliva production and elevated concentrations of albumin in the saliva (Fig. 5d, e). To further probe the impact of tricellulin deficiency on the integrity of the acinar epithelial barrier, we conducted an in vivo paracellular permeability assay using rhodamine B-labeled dextran (*Mr*, 40 kD, termed as RD40) as a macromolecular tracer. Following a 30-min exposure to lipopolysaccharide (LPS, administered at 5 mg/kg body weight), a greater accumulation of RD40 signal was observed within the acinar lumens of *Tric*^*CKO*^ mice after pilocarpine administration, contrasting with the response in *Tric*^*flox/flox*^ controls (Fig. 6). These findings provide direct evidence that the absence of tricellulin in acinar epithelial cells triggers a decline in saliva secretion and facilitates macromolecular leakage in salivary glands.

### Expression of tricellulin is downregulated by IFN-γ in SMG-C6 cells

**Fig. 7:**
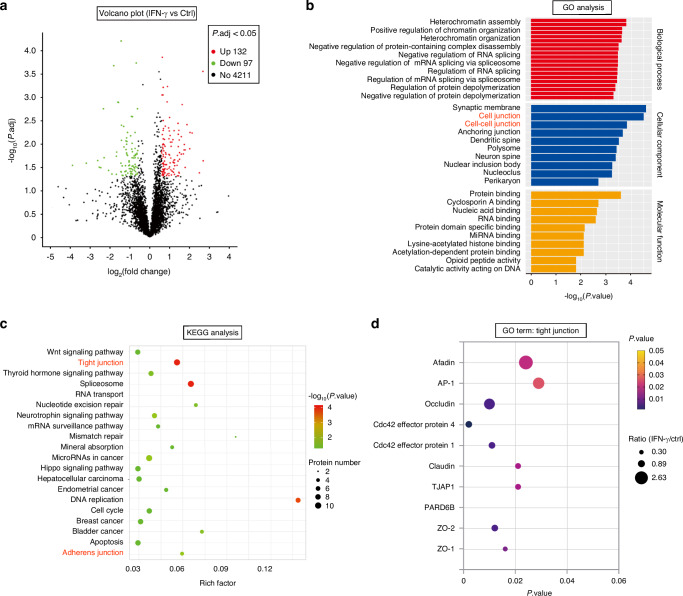
The phosphorylated protein profiles of rat submandibular gland epithelial polarized cell line SMG-C6 treated with interferon-γ (IFN-γ) for 30 min and controls were screened using proteomics. **a** Volcano plot displaying differential expression of 229 proteins in IFN-γ-treated and control groups (*n* = 3). Proteins upregulated (ratio of fold change | FC| ≥ 1.5) in treated groups are shown in red. Proteins downregulated (ratio of fold change | FC| ≤ 0.67) in treated groups are shown in green. **b** The Gene Ontology Enrichment (GO) analysis was performed to investigate cellular biological processes. Cellular components shown in red are related to cell junctions. **c** The 20 most enriched KEGG pathways for the differentially expressed proteins in IFN-γ-treated SMG-C6 cells and controls. Pathways shown in red are related to tight junction and adherens junction pathway. **d** The differentially expressed proteins in the GO term “Tight junction” in control and IFN-γ-treated groups. The 10 most enriched tight junction-related proteins, such as afadin, AP-1, occludin, Cdc42 effective protein, claudin, TJAP1, PARD6B, ZO-1, and ZO-2, were phosphorylated in SMG-C6 cells treated with IFN-γ. TJAP1 tight junction-associated protein 1, PARD6B PAR-6 family cell polarity regulator beta, AP-1 activating protein 1

**Fig. 8:**
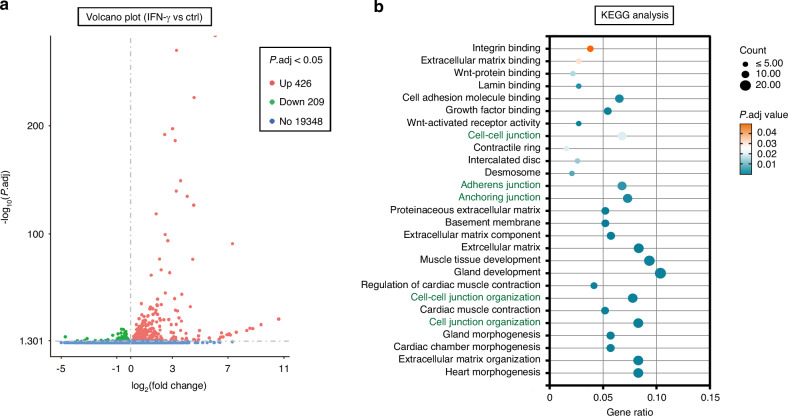
High-throughput RNA sequencing was performed in rat submandibular gland epithelial polarized cell line SMG-C6 with or without interferon-γ (IFN-γ) stimulation for 24 h. **a** Volcano plot displaying differential expression of 635 genes in IFN-γ-treated and control groups (*n* = 4). Proteins upregulated (ratio of fold change | FC| ≥ 1.0) in treated groups are shown in red. Proteins downregulated (ratio of fold change | FC| ≤ 0.5) in IFN-γ-treated group are shown in green. **b** The 20 most enriched KEGG pathways for the down-expressed genes in IFN-γ-treated and control groups. Pathways shown in green are related to cell junction, adherens junction, anchoring junction, and cell junction organization pathways

**Fig. 9:**
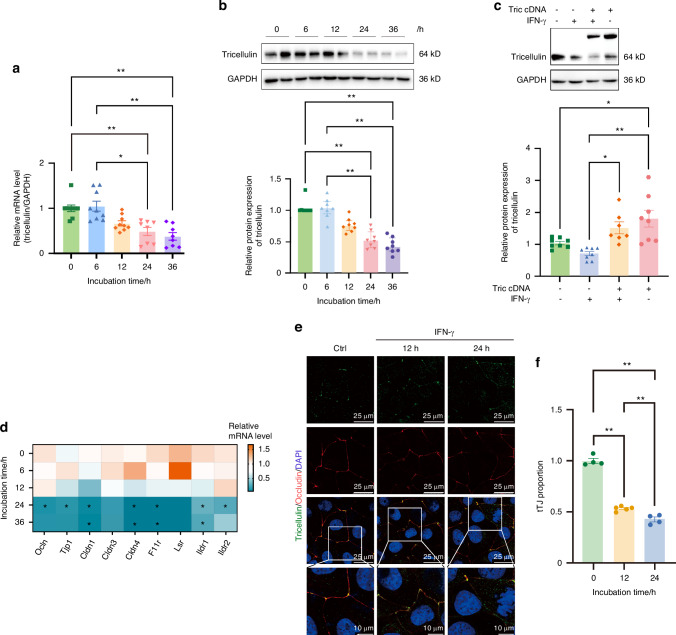
The alteration of tricellulin in rat submandibular gland epithelial polarized cell line SMG-C6 treated with interferon-γ (IFN-γ). **a**, **b** The mRNA (**a**) and protein (**b**) expression of tricellulin induced by IFN-γ with different times. *n* = 7–8. **c** Overexpression of tricellulin in SMG-C6 cells treated with IFN-γ for 24 h. **d** The mRNA levels of tight junction proteins in IFN-γ-treated and control groups. *n* = 4. **e** The co-staining of tricellulin (green) and occludin (red) in SMG-C6 cells. Cell nuclei were stained with DAPI (blue). The enlarged images (bar: 10 µm) were derived from boxes in the upper panels (bar: 25 µm). Arrows pointed to the distribution of tricellulin. **f** The semiquantitative tricellulin expression in IFN-γ-treated and control groups according to enlarged images (**e**). The tricellular tight junction (tTJ) proportion was the ratio of fluorescent signals of tricellulin at tTJ to that in the whole cell. Analysis was performed by using Kruskal–Wallis’ test (**a**–**d**, **f**) where *P < 0.05 and **P < 0.01. The data are presented as means ± SEM (**a**–**c**, **f**), or means (**d**)

IFN-γ plays a critical role as an inflammatory cytokine, inciting dysfunction in salivary gland epithelial cells, and its levels are prominently elevated in the salivary glands of SS patients.^15,16^ To emulate the inflammatory milieu characteristic of SS, the rat SMG epithelial polarized cell line, SMG-C6, was exposed to IFN-γ. Proteomics analysis was employed to screen for differential phosphorylation profiles in SMG-C6 cells following 30 min of IFN-γ treatment versus controls. This analysis identified 229 differentially phosphorylated proteins (with a ratio of fold change ≥1.5 or ≤0.67, *P* < 0.05), among which 132 proteins were upregulated and 97 were downregulated in the IFN-γ-treated group (Fig. 7a). Gene Ontology (GO) analysis of cellular components revealed an enrichment of proteins associated with cell junctions and cell-cell junctions (Fig. 7b). Moreover, KEGG pathway analysis disclosed that TJ and AJ-related proteins were prominently featured among the top 20 pathways (Fig. 7c). Focusing on the term “Tight junction” within the GO-enriched categories, we identified afadin, activating protein 1, occludin, Cdc42 effector protein 1 and 4, claudin, tight junction-associated protein 1 (TJAP1), PAR-6 family cell polarity regulator beta (PARD6B), ZO-1, and ZO-2 (Fig. 7d). However, tricellulin was absent from the list of phosphorylated proteins, suggesting that IFN-γ stimulation does not elicit rapid phosphorylation modifications of tricellulin. Subsequently, high-throughput RNA sequencing was performed in SMG-C6 cells treated with or without IFN-γ for 24 h. This analysis uncovered 635 differentially expressed genes (with a ratio of fold change ≥ 1 or ≤ 0.5, *P* < 0.05), of which 426 genes were upregulated and 209 were downregulated (Fig. 8a). The downregulated genes were strongly associated with cell-cell junction, cell-cell junction organization, cell junction organization, adherens junction, and anchoring junction (Fig. 8b). Validation of the sequencing results revealed that IFN-γ significantly suppressed tricellulin mRNA and protein expression at both 24 h and 36 h post-treatment (Fig. 9a, b). Overexpression of tricellulin partially mitigated the inhibitory effect of IFN-γ on tricellulin protein expression (Fig. 9c). Additionally, the mRNA levels of occludin, ZO-1, claudin-1, claudin-4, JAM-1, ILDR1, and ILDR2 were decreased by IFN-γ stimulation at 24 h, and the reduction persisted for claudin-1, claudin-4, JAM-1, and ILDR1 at 36 h (Fig. 9d). Immunofluorescence imaging demonstrated that tricellulin was primarily localized as puncta at tricellular contacts in untreated cells, whereas tricellulin was redistributed to bicellular junctions upon IFN-γ treatment for 12 h and 24 h (Fig. 9e, f).

**Fig. 10:**
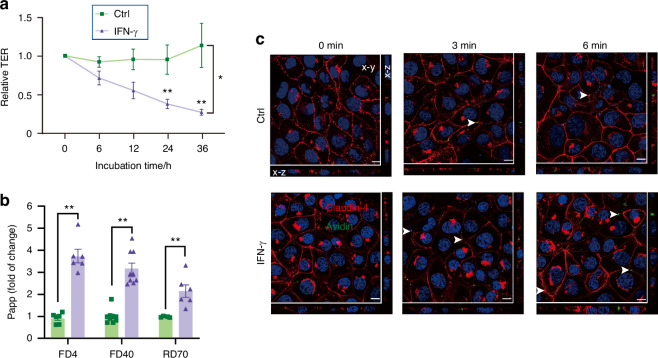
The changes of the epithelial barrier function determined by transepithelial electrical resistance (TER) measurement (**a**) and by in vitro paracellular permeability assays (**b**) using 4 kDa and 40 kDa fluorescein isothiocyanate (FITC)-labeled dextran (FD4 and FD40) and 70 kDa rhodamine B-labeled dextran (RD70) in IFN-γ-treated and control groups. *n* = 5–9. SMG-C6 cells were seeded at a low density (2 × 10^4^ cells/cm^2^) in Costar 24-well Transwell chambers. For TER measurement (**a**), the values showed the changes of ion transports induced by IFN-γ with different times. For paracellular permeability assays (**b**), the flux of tracers showed the changes of molecules with different weights induced by IFN-γ treatment for 24 h. The apparent permeability coefficient (Papp) was calculated as the increase in the tracer amount per time and per filter area. **c** The transport of avidin-FITC (green) in IFN-γ-treated and control groups. Claudin-4 (red) was used to line the cell membrane. Cell nuclei were stained with DAPI (blue). Arrows pointed to the flux of avidin. Bar: 10 µm. Analysis was performed by using 2-way ANOVA (**a**) and unpaired two-tailed *t* test (**b**) where *P < 0.05 and **P < 0.01. The data are presented as means ± SEM (**a**, **b**)

To explore the pivotal role of tricellulin in preserving barrier integrity in salivary gland epithelial cells, transepithelial electrical resistance (TER) measurement and paracellular permeability assay, two typical assays to evaluate barrier function, were performed in SMG-C6 cells. In our experiments with SMG-C6 cells, IFN-γ stimulation was found to significantly reduce TER values in a time-dependent manner (Fig. 10a), indicative of compromised epithelial barrier function. In addition, the permeation of fluorescein isothiocyanate (FITC)-labeled dextran (*Mr*, 4 and 40 kDa, termed as FD4 and FD40) and rhodamine B-labeled dextran (*Mr*, 70 kD, termed as RD70) was notably higher in IFN-γ-treated cells compared to their control cells (Fig. 10b). To precisely delineate the route through which macromolecules traverse, we conducted real-time monitoring of avidin-FITC flux. Our results indicated that in control cells, avidin-FITC predominantly passed through the points of tricellular contact, a phenomenon that was markedly exacerbated by IFN-γ stimulation (Fig. 10c, Supplemental Video). These findings support the hypothesis that the downregulation of tricellulin by IFN-γ accelerates the passage of macromolecules, particularly via the tricellular pathways, thus compromising the barrier integrity of salivary gland epithelial cells.

### JAK/STAT1/miR-145 axis mediates the effect of IFN-γ on tricellulin expression

**Fig. 11:**
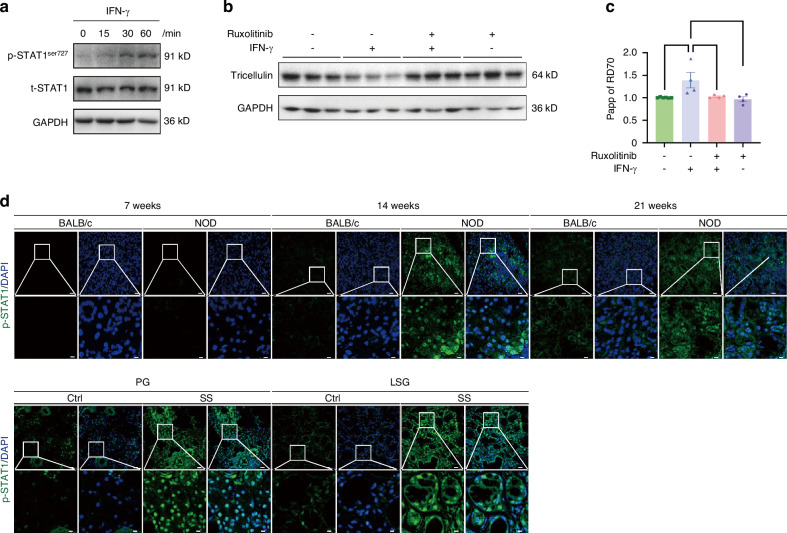
The regulatory mechanism of interferon-γ (IFN-γ) on tricellulin in salivary glands. **a** The activation of signal transducer and activator of transcription 1 (STAT1) induced by IFN-γ at different times (0, 30, and 60 min) in rat submandibular gland epithelial polarized cell line SMG-C6. **b**, **c** The changes of the protein expression of tricellulin (**b**) and epithelial barrier function (**c**) regulated by silencing tyrosine-protein kinase (JAK)/STAT1 pathway in SMG-C6 cells treated with IFN-γ for 24 h. Ruxolitinib is regarded as the silencer for JAK. SMG-C6 cells were seeded at a low density (2 × 10^4^ cells/cm^2^) in Costar 24-well Transwell chambers. The apparent permeability coefficient (Papp) was calculated as the increase in the tracer amount per unit time and per filter area. 70 kDa rhodamine B-labeled dextran (RD70) was used in the epithelial permeability assay. *n* = 4–6. **d** The immunostaining of phospho-STAT1 (p-STAT1) in Sjögren’s syndrome (SS) animal models and salivary gland biopsies from SS patients. Cell nuclei were stained with DAPI (blue). The enlarged images (bar: 8 µm) were derived from boxes in the upper panels (bar: 25 µm). PG parotid gland. SLG sublingual gland. Analysis was performed by using one-way ANOVA (**c**) where *P < 0.05 and **P < 0.01. The data are presented as means ± SEM

We next sought to elucidate the potential signaling pathway regulating tricellulin expression in salivary gland epithelial cells. The phosphorylation of signal transducer and activator of transcription 1 (STAT1) at Ser727 was significantly increased after 30 min and 60 min of IFN-γ treatment (Fig. 11a, Supplemental Fig. 4a). Pretreatment with ruxolitinib, a JAK/STAT1 pathway inhibitor, effectively reversed the IFN-γ-induced downregulation of tricellulin expression (Fig. 11b, Supplemental Fig. 4b). Moreover, the increased flux of RD70 induced by IFN-γ was also attenuated by pretreatment with ruxolitinib (Fig. 11c). Immunofluorescence staining revealed higher intensities of phosphorylated STAT1 in the SMGs of 14- and 21-week-old NOD mice, as well as in the PG and LSG of SS patients (Fig. 11d). These observations imply that IFN-γ mediates the downregulation of tricellulin by activating the JAK/STAT1 signaling pathway. Toll-like receptors (TLRs) play crucial roles in the innate immune system by recognizing pathogen-associated molecular patterns derived from various microbes. As summarized in a recent review, several TLRs, including TLR2, TLR3, TLR4, TLR7, and TLR9, are involved in the pathogenesis of SS by modulating inflammatory factor expression, B cell maturation, and salivary epithelial cell apoptosis.^28^ Accordingly, we performed qPCR to detect the changes of these above mentioned TLRs in the SMGs of BALB/c and NOD mice. Compared to age-matched BALB/c mice, the level of TLR9 was upregulated in 7-week-old NOD mice, the levels of TLR4, TLR7, and TLR9 were significantly upregulated in 14-week-old NOD mice, and the levels of TLR2 and TLR4 were elevated in 21-week-old NOD mice (Supplemental Fig. 4e). The precise mechanism of TLR receptor pathway in the occurrence of SS will be explored in the future studies.

**Fig. 12:**
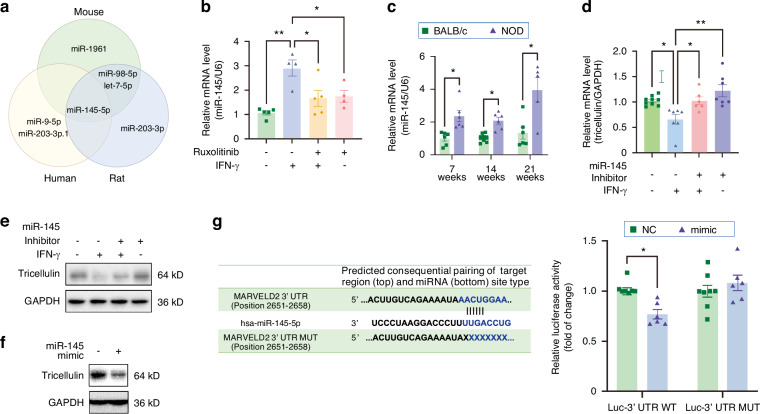
MicroRNA-145 (miR-145) downregulates the expression of tricellulin during Sjögren’s syndrome (SS). **a** The prediction of miRNAs targeting tricellulin mRNA by using TargetScan website among human, mouse and rat species. **b**, **c** The changes of miR-145 expression in rat submandibular gland epithelial polarized cell line SMG-C6 treated with interferon-γ (IFN-γ) for 24 h (**b**) and non-obese diabetic (NOD) mice (**c**). *n* = 4–5 (**b**) and *n* = 6–11 (**c**). **d**, **e** The mRNA (**d**) and protein expression (**e**) of tricellulin regulated by miR-145 inhibitor in SMG-C6 cells treated with IFN-γ for 24 h and controls. *n* = 6–8. **f** The regulation of miR-145 mimic on tricellulin expression in SMG-C6 cells. **g** The binding between miR-145 and tricellulin mRNA 3’ UTR by dual-luciferase assay. n = 6-8. Analysis was performed by using one-way ANOVA (**b**, **d**) and unpaired two-tailed *t* test (**c**, **g**), where *P < 0.05 and **P < 0.01. The data are presented as means ± SEM

Many previous studies have underscored the involvement of microRNAs (miRNAs) in the modulation of TJs. Consequently, we endeavored to identify a specific miRNA that could regulate tricellulin expression in salivary glands. Employing TargetScan prediction tool to predict the miRNAs that bind to the 3’ untranslated region (UTR) of tricellulin mRNA covering human, mouse, and rat species,^29^ we pinpointed miR-145-5p (referred to as miR-145 hereafter) as a potential regulator (Fig. 12a). The expression of miR-145 was observed to escalate in response to 24 h of IFN-γ stimulation in SMG-C6 cells, whereas this phenomenon was abrogated by ruxolitinib pretreatment (Fig. 12b). The level of miR-145 was also found to be higher in 7-, 14-, and 21-week-old NOD mice when compared to age-matched BALB/c mice (Fig. 12c). Further experimentation revealed that pretreatment with a miR-145 inhibitor attenuated the IFN-γ-induced decline in tricellulin mRNA and protein expression (Fig. 12d, e), whereas a miR-145 mimic led to a suppression of tricellulin protein levels (Fig. 12f). A dual-luciferase reporter assay confirmed that miR-145 binding to the tricellulin 3’ UTR reduced luciferase activity, an effect that was negated when the putative binding sites were mutated (Fig. 12g). Taken together, these results substantiate that miR-145 acts as a direct mediator of tricellulin expression under the influence of the IFN-γ/JAK/STAT1 axis.

### Enhancement of TJ barrier function and restorage of tricellulin expression ameliorates hyposalivation in NOD mice

**Fig. 13:**
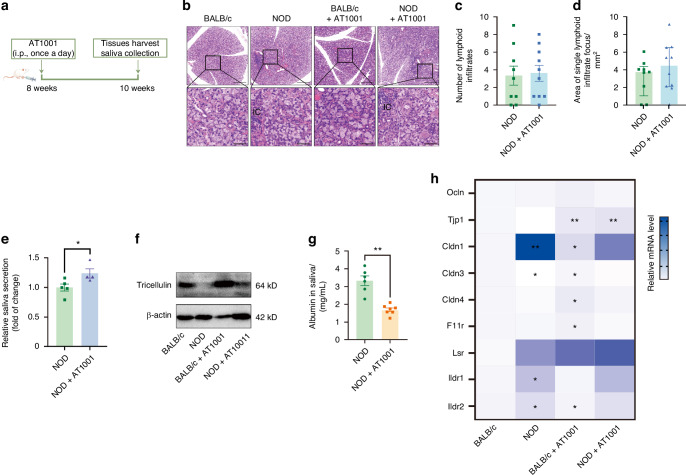
The regulation of hyposalivation by sealing tight junction in non-obese diabetic (NOD) mice. **a** Schematic representation of experimental design in NOD mice. Intraperitoneal injections of tight junction sealer AT1001 were administered into 8-week-old NOD mice once a day for two weeks. **b** Hematoxylin and eosin (H&E) staining of SMGs from NOD mice with or without AT1001 treatment. The enlarged images (bar: 50 µm) were derived from boxes in the upper panels (bar: 200 µm). IC infiltrating cells. **c**, **d** The number of lymphocytic focus (**c**) and the size of single infiltrate center (**d**) according to (**c**). *n* = 8–10. **e** The stimulated saliva secretion of NOD mice with or without AT1001 treatment. *n* = 4–5. **f** The influence of AT1001 on the expression of tricellulin in SMGs of NOD mice. **g** The level of albumin in NOD mice with or without AT1001 treatment. *n* = 6–7. **h** The mRNA expressions of tight junction proteins of SMGs in NOD mice with or without AT1001 treatment. *n* = 5–6. Analysis was performed by using unpaired two-tailed *t* test (**c**–**e**), Mann-Whitney’s test (**g**) and Kruskal-Wallis’ test (**h** compared to NOD group) where *P < 0.05 and **P < 0.01. The data are presented as means ± SEM (**c**–**e**, **g**) and means (**h**)

**Fig. 14:**
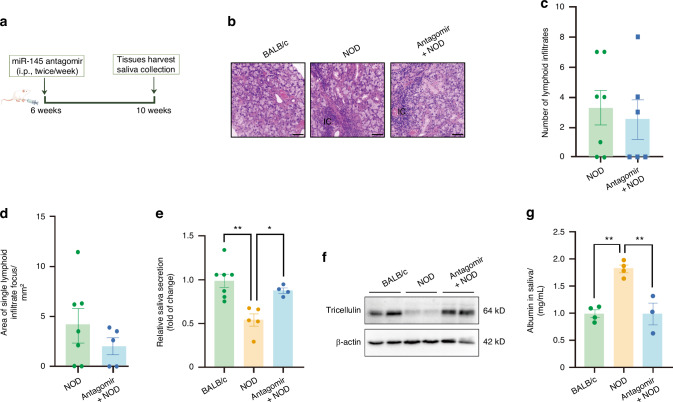
The regulation of hyposalivation by inhibiting microRNA-145 (miR-145) in non-obese diabetic (NOD) mice. **a** Schematic representation of experimental design in NOD mice. Intraperitoneal injections of miR-145 antagomir (100 nmol per mouse) were administered into 6-week-old NOD mice twice a week for one month. **b** Hematoxylin and eosin (H&E) staining of SMGs of NOD mice with or without miR-145 antagomir. bar = 50 μm. **c**, **d** The number of lymphocytic focus (**c**) and the size of single infiltrate center (**d**) according to (**b**). *n* = 8–10. **e** The stimulated saliva secretion of NOD mice with or without miR-145 antagomir. *n* = 4–7. **f** The influence of miR-145 antagomir on the expression of tricellulin in SMGs of NOD mice. **g** The level of albumin in NOD mice with or without AT1001 treatment. *n* = 6–7. Analysis was performed by using unpaired two-tailed *t* test (**c**, **d**) and one-way ANOVA (**c**, **e**) where *P < 0.05 and **P < 0.01. The data are presented as means ± SEM (**c**–**e**, **g**)

Finally, we examined whether enhancing epithelial barrier function through the application of the TJ sealer, AT1001 (larazotide acetate), could confer therapeutic effects on treating hyposalivation. Histological morphology analysis revealed that the structural integrity of SMGs in 10-week-old BALB/c and NOD mice remained unaltered regardless of AT1001 treatment (Fig. 13a, Supplemental Fig. 4f). While AT1001 did not mitigate the extent of inflammatory infiltration, it notably restored saliva secretion in NOD mice (Fig. 13b–e). Meanwhile, AT1001 treatment resulted in a recovery of tricellulin protein expression in the SMGs of NOD mice, in contrast to those treated with PBS, alongside a marked reduction in saliva albumin levels (Fig. 13f, g, Supplemental Fig. 4g). Additionally, the abnormal expression of other TJ components, such as claudin-1, claudin-3, ILDR1, and ILDR2, was normalized in the SMGs of NOD mice following AT1001 intervention (Fig. 13h). To specifically investigate the role of tricellulin in salivation, we further administered miR-145 antagomir via intraperitoneal injection into 6-week-old NOD mice (Fig. 14a). Although no significant improvement in inflammation was observed in the SMGs of 10-week-old NOD mice, miR-145 antagomir significantly alleviated hyposalivation and restored tricellulin expression in NOD mice (Fig. 14b–f, Supplemental Fig. 4h, i). Furthermore, the elevated albumin levels in the saliva of NOD mice were attenuated following miR-145 antagomir treatment (Fig. 14g). These results suggest that targeting TJs, with a particular focus on tricellulin, is a promising therapeutic strategy for curing hyposalivation in SS.

## Discussion

**Fig. 15:**
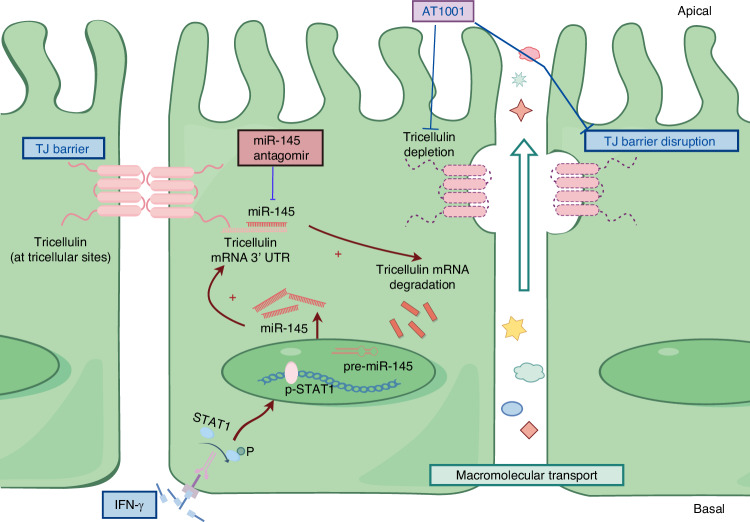
A schematic diagram illustrates a proposed mechanism of interferon-γ (IFN-γ)-induced downregulation of tricellulin in salivary epithelial cells. IFN-γ leads to the phosphorylation of signal transducer and activator of transcription 1 (STAT1) and then induces the upregulation of microRNA-145 (miR-145) which combines with tricellulin mRNA 3’ UTR and inhibits the expression of tricellulin. This would then cause the dysfunction of acinar epithelial barrier and the increased transports of macromolecules through paracellular pathway due to the loss of tricellulin in salivary glands, thereby resulting in hyposalivation. The schematic diagram is designed by Figdraw

Currently, the understanding of hyposecretion in exocrine glands and its underlying mechanisms remains enigmatic, presenting a significant obstacle to the development of effective early treatments for SS in clinical settings. Our investigation elucidates the diminution of tricellulin in salivary gland acinar epithelial cells as a pivotal and early event, directly contributing to hyposalivation in SS. Mechanistically, we delineate the JAK/STAT1/miR-145 pathway as a mediator of IFN-γ-induced downregulation of tricellulin. Furthermore, our data demonstrate that fortifying disrupted epithelial TJs and restoring tricellulin expression can effectively curb the progression of SS, as illustrated in the conceptual diagram (a scheme in Fig. 15). From this perspective, targeting tricellulin to enhance epithelial barrier function emerges as a promising therapeutic strategy for SS.

Our study reveals a significant finding that tricellulin deficiency is an early event in SS pathogenesis, preceding hyposalivation and correlating with aberrant transport of macromolecules in salivary glands. It is well-known that TJs constitute an intercellular barrier that protects against the free transport of materials and even cells.^30^ Unlike the classical bTJ components, tTJs are dominantly expressed at the sites where three cells meet and form a “central tube” that has a larger diameter than the bicellular route.^5,6^ Mutations in the tricellulin gene underlie nonsyndromic deafness (DFNB49), a human hereditary disease, due to impaired interaction with TJ scaffolding proteins ZO-1 to -3.^31^ Knockdown of occludin or lipolysis-stimulated lipoprotein receptor (LSR) leads to the mislocalization of tricellulin from tricellular junctions to bicellular junctions.^32,33^ Our earlier work reported that tricellulin modulates macromolecular transport and alters bTJ structure in salivary gland epithelial cells.^10^ These findings underscore the critical role of tricellulin in controlling macromolecular transport across epithelia and suggest potential impacts on other TJs. In this study, we observed markedly reduced tricellulin expression in salivary glands of SS patients. Using NOD mice as models for disease progression, we pinpointed the loss of tricellulin as an early phenomenon. Measuring the levels of albumin as an indicator for macromolecular paracellular transport, we revealed heightened albumin flux in the saliva of NOD mice concurrent with tricellulin loss. Given the altered expression of other TJ components in SMGs of NOD mice, we generated salivary gland acinar cell-specific tricellulin knockout mice to directly identify the effects of tricellulin. Compared to control *Tric*^*flox/flox*^ mice, *Tric*^*CKO*^ mice exhibited reduced saliva secretion, widened TJs, and increased leakage of paracellular tracers in SMGs. Our data indicate that tricellulin deficiency contributes to the abnormal secretory patterns observed in SS. This finding elucidates why various large molecules, such as β2-microglobulin, lactoferrin, soluble sialic acid-binding immunoglobulin-like lectin-5, cytokines (e.g., IL-17, IL-6, IL-10, and TNF-α), antibodies against muscarinic acetylcholine receptor M3, calprotectin, C-reactive protein, and albumin, are elevated in the saliva of SS patients relative to healthy individuals.^34–38^ The potential use of these salivary biomarkers as non-invasive diagnostic indicators for SS may derive from the absence of tricellulin in epithelial cells.

An additional important finding from our research is the identification of the signaling pathway that governs the expression of tricellulin in salivary glands. In SS, Th1 lymphocytes and their secreted cytokines, notably IFN-γ and TNF-α, predominate as inflammatory mediators in salivary glands.^39^ Prior investigations have shown that IFN-γ and/or TNF-α regulate the expression and distribution of TJ components by affecting the myosin II-dependent vesicles on the apical membranes and reorganizing cytoskeleton, lead to the internalization of occludin, claudin-1, and JAM-1 through the stimulation of Ras homologue family member A (RhoA)/RhoA kinase, and downregulate the expression of ZO-1 and claudin-10 through NF-κB and JAK/STAT signaling, respectively.^40–45^ In our current study, we found that stimulation with IFN-γ significantly reduced both the mRNA and protein expression of tricellulin in SMG-C6 cells. Furthermore, inhibiting the JAK/STAT1 pathway effectively abrogated the IFN-γ-induced suppression of tricellulin expression and the resulting increase in macromolecular leakage.

MiRNAs are a group of endogenous non-coding RNAs composed of ~22 nucleotides, subtly coordinating cellular function by binding to the 3’ UTR of target mRNAs and repressing the expression of targeted genes.^46^ Previous studies have revealed the role of miRNAs in the regulation of TJs. For example, miR-21 has been observed to be upregulated and contributes to increased permeability in intestinal and mucosal tissues in patients diagnosed with ulcerative colitis and Crohn’s disease.^46,47^ The miR-200c-mediated degradation of occludin mRNA is implicated in the IL-1β-induced disruption of intestinal epithelial TJs.^47^ High glucose conditions have been shown to decrease paracellular permeability by inhibiting miR-22-3p/SP-1-mediated claudin-1 and claudin-3 expressions.^48^ Exosomal miR-23b-3p derived from cancer cells leads to increased vascular permeability and a reduction in the expression of occludin, ZO-1, and claudin-1 in salivary adenoid cystic carcinoma.^49^ In the present study, miR-145 was predicted to specifically target tricellulin mRNA through TargetScan, and verified to negatively regulate tricellulin expression. The dual-luciferase reporter assay further confirmed that miR-145 binds to the 3’ UTR region of tricellulin mRNA. Although a previous study documented lower levels of miR-145 in LSGs from SS patients,^50^ our findings revealed an elevated level of miR-145 in SMGs of NOD mice and in SMG-C6 cells stimulated with IFN-γ. This discrepancy could potentially be attributed to differences in disease stage between the models utilized, and additional research is warranted to clarify this inconsistency. Collectively, these data suggest that IFN-γ orchestrates the downregulation of tricellulin expression in salivary glands through a mechanism involving the JAK/STAT1/miR-145 axis.

Ultimately, in light of the evidence elucidating the causative link between tricellulin insufficiency and hyposalivation in SS, we administered AT1001 to augment TJ barrier function and promote tricellulin expression. AT1001, a synthetic peptide, has been shown to consolidate TJs at the cellular membrane level, thereby reinforcing barrier integrity.^51^ Its therapeutic potential has been explored across numerous TJ-associated disease models, including celiac disease, type 1 diabetes, and respiratory diseases.^51–53^ We previously reported the effectiveness of AT1001 in mitigating salivary gland fibrosis by enhancing microvascular endothelial function. Within the scope of this study, we observed that AT1001 treatment substantially alleviated hyposecretion and reduced macromolecular leakage in NOD mice. These findings imply that bolstering TJ barrier function and restoring tricellulin expression confer benefits to SMGs impaired in NOD mice. Moreover, considering that the effect of AT1001 is not restricted to tricellulin, we treated NOD mice with miR-145 antagomir to specifically recover the expression of tricellulin. Results showed restoring tricellulin expression by miR-145 antagomir significantly alleviated hyposalivation and the leakage of macromolecules in NOD mice, suggesting that tricellulin may serve as a promising target for the treatment of SS.

In summary, the findings of this study highlighted the crucial role of epithelial tricellulin in maintaining barrier function and its dysfunction in the pathogenesis of SS. Our results demonstrated that tricellulin expression is suppressed by IFN-γ via the JAK/STAT1/miR-145 signaling pathway. Moreover, we established that the restoration of TJ integrity and the re-expression of tricellulin are instrumental in alleviating hyposalivation. Consequently, targeting acinar epithelial tricellulin represents a promising therapeutic strategy for addressing hyposecretion and macromolecular leakage in SS.

## Materials and methods

### Reagents and antibodies

Pilocarpine, tamoxifen, fluorescein isothiocyanate (FITC)-labeled dextran (*Mr*, 4 and 40 kDa), rhodamine B-labeled dextran (*Mr*, 40 and 70 kDa), EZ-link NHS-LC-LC-Biotin, avidin-FITC (*Mr*, 68 kDa), IFN-γ, and cell culture constituents were purchased from Sigma-Aldrich. Ruxolitinib, rhodamine B labeled phalloidin and larazotide acetate (also known as AT1001) were purchased from MedChemExpress. Antibodies against tricellulin, CD3, and CD4 were from Invitrogen and Proteintech. Antibodies against phospho-STAT1 (p-STAT1) and total STAT1 (t-STAT1) were from Cell Signaling Technology. Antibodies against occludin and claudin-4 were from Invitrogen.

### Public datasets and bioinformatics analysis

The gene expression matrices of GSE173808 and GSE208260 datasets were obtained from the Gene Expression Omnibus (GEO) (www.ncbi.nlm.nih.gov/geo/). Differentially expressed genes were analyzed with “voom” method of limma R package (version 3.56.2). Genes with adjust.*P*.value < 0.05 and | log2 Fold Change| ≥ 1.0 were regarded as significant change. The expression changes of TJ genes were visualized using a dot plot generated with the ggplot2 R package (version 3.4.2).

GSEA analysis was performed using the ClusterProfiler R package (version 4.8.2). The ontology gene set (c5.all.v7.0) from the Molecular Signatures Database (MSigDB) was used for annotation. Terms associated with TJs were extracted, and those with an adjust.*P*.value < 0.05 were considered significantly enriched.

### Human salivary gland tissue collection

Our study exclusively examined female patients because middle-aged and elderly women are the most commonly affected group with SS. LSG biopsies were obtained from 6 female patients with SS and one PG biopsy was obtained from another female patient with SS. LSGs and PGs from patients who underwent mucocele resection and were confirmed to be histologically normal were used as controls. The relevant information for the SS patients is listed in the Supplemental Table 1. The research protocol was approved by the Peking University Institutional Review Board (PKUSSIRB-201631139), and all patients signed an informed consent document prior to tissue collection.

### Animal models and saliva collection

Our study examined female NOD mice because female mice have a higher prevalence of spontaneous inflammation of the salivary glands and are more prone to dry mouth compared to male mice. Seven-, 14-, and 21-week-old female NOD mice and age-matched BALB/c mice (as control groups) were purchased from Gempharmatech Cooperation, and only NOD mice with blood glucose ≤ 250 mg/dL were included in the experiments. After anesthesia, whole saliva was collected for 10 min from oral cavity following pilocarpine stimulation (10 µg/g body weight, i.p.). All experimental procedures were approved by the Ethics Committee of Animal Research, Peking University Health Science Center (LA2019220), and complied with the Guide for the Care and Use of Laboratory Animals (NIH Publication No. 85-23, revised 1996).

To explore the role of tricellulin in saliva secretion, the salivary gland acinar cell-specific tricellulin conditional knockout mice were generated by intercrossing *Tric*^*flox/flox*^ mice with *Aqp5-CreER*^*T2*^ mice as previously described.^54^ Genotyping was performed using tail samples from mice at 2-3 weeks of age. Six- to 7-week-old male *Tric*^*flox/flox*^*; Aqp5-CreER*^*T2*^ (referred to as *Tric*^*CKO*^) mice and their littermate *Tric*^*flox/flox*^ mice were intraperitoneally injected with tamoxifen (75 mg/kg body weight, Sigma-Aldrich) for seven days, and the experiments were performed after a two-week waiting period. To further explore the function of TJs in saliva secretion, AT1001,^51^ which is a first-in-class TJ sealer, was intraperitoneally injected into 8-week-old female NOD and BALB/c mice for two weeks. To gain further insights into the regulation of tricellulin, we administered miR-145 antagomir (100 nmol per mouse, RiboBio) via intraperitoneal injection to 6-week-old NOD mice twice a week for a period of four weeks.

### Histological and immunofluorescence staining

Paraffin or frozen sections of SMGs were stained with hematoxylin and eosin (H&E), and morphological changes were observed under a light microscope. SMG tissue sections (7 µm) were fixed in cold paraformaldehyde, blocked with 3% bovine serum albumin, stained with primary antibodies at 4 °C overnight, and then incubated with Alexa 594- or Alexa 488-conjugated secondary antibodies at 37 °C for 2 h. Nuclei were stained with 4’, 6-diamidino-2-phenylindole (DAPI). Fluorescence images were captured using a confocal microscope (Leica Stellaris 8).

### qPCR

Following the isolation of total RNA, cDNA was synthesized using HiScript III 1st Strand cDNA Synthesis Kit (Vazyme). The primers are shown in the Supplemental Table 2. qPCR was performed using a Thermo PikoReal PCR System (ThermoFisher Scientific). The level of miRNA was examined using miDETECT A Track miRNA qPCR Starter Kit (RiboBio).

### Western blot

The samples were homogenized in RIPA buffer (Thermo Fisher Scientific) and sonicated for 21 s (3 s on and 3 s off), and then centrifuged at 12 000 × *g* for 10 min at 4 °C. The supernatant was collected, and the protein concentration was determined by the Bradford method (Solarbio). Equal amounts of proteins (20–40 µg) were separated on a 10% SDS-PAGE gel at a constant voltage 120 V for approximately 1.5 h and transferred onto a polyvinylidene difluoride membrane at a constant current of 200 mA for 2–3 h. The membranes were blocked with 5% non-fat milk for 2 h at room temperature, probed with primary antibodies at 4 °C overnight, and incubated with horseradish peroxidase (HRP)-conjugated secondary antibodies for 2 h at room temperature. Immunoreactive bands were visualized using enhanced chemiluminescence (Biodragon), and their densities were quantified using Image J software (National Institutes of Health).

### ELISA

The level of albumin in the saliva harvested from mice was measured by ELISA according to the manufacturer’s protocol (Abcam). An intraperitoneal injection of pilocarpine (10 μg/g body weight) was administered into mice to stimulate saliva secretion and saliva samples were collected for 10 min. Subsequently, primary saliva samples were centrifuged at 800 *×* *g* for 10 min and then diluted 4 000-fold using Diluent *N* to achieve an appropriate dilution. The whole assay was performed at room temperature (20–25 °C).

### Transmission electron microscopy

The freshly harvested mouse SMG tissues were cut into 1 mm^3^ and fixed in precooled 2.5% glutaraldehyde. Ultrathin sections were stained with uranyl acetate and lead citrate and then observed using a transmission electron microscope (Hitachi). The distances between neighboring TJs were measured using Image J software (National Institutes of Health).

### Cell culture

Rat SMG epithelial polarized cell line SMG-C6, gifted by Prof. David O. Quissell, was cultured at 37 °C in a 5% CO_2_ incubator using the same cell medium as previously reported.^10^ The human embryonic kidney cell line 293 T was purchased from the American Type Culture Collection (ATCC CRL-3216) and cultured in DMEM supplemented with 10% fetal bovine serum.

### Proteomics analysis

The proteins of three SMG-C6 cell samples after IFN-γ (final concentration, 50 ng/mL) treatment for 30 min and three controls were collected. The protein concentration was determined using a suitable assay. The proteins were subjected to capillary electrophoresis-mass spectrometry (CE-MS), protease digestion, and phosphorylation peptide enrichment. The digested peptides were then analyzed by liquid chromatography-tandem mass spectrometry (LC–MS/MS). The MaxQuant software was used to process the obtained RAW files from the mass spectrometry data. The data were searched against the UniProt rat database (uniprot_rat_36135_20200211.fasta) to identify proteins and phosphorylation sites. Statistical analysis was performed to identify differentially phosphorylated sites. Bioinformatic analysis was conducted to investigate the functions and pathways involved.

### RNA sequencing

Four SMG-C6 cell samples treated with IFN-γ treatment for 24 h and four control samples were used for RNA-seq experiments, which were performed by Novogene. Differential gene expression analysis was conducted using the the DESeq2 software (version 1.20.0) for samples with biological replicates. Genes with an adjust.*P*.value ˂ 0.05 were considered differentially expressed. For samples without biological replicates, the edgeR method was used, with the corrected *P* value and | log_2_ Fold Change | as thresholds for significant differential expression. Statistical enrichment of differentially expressed genes in KEGG pathways was analyzed using the ClusterProfiler (version 3.4.4) software.

### Transepithelial electrical resistance (TER) measurement

SMG-C6 cells were seeded at a low density (2 × 10^4^ cells per cm^2^) in Costar 24-well Transwell chambers (filter pore size: 0.4 µm, filter area: 0.33 cm^2^). Cells were grown to form a confluent monolayer, and then TER was measured at 37 °C using an epithelial Volt/Ohm meter (EVOM2, World Precision Instruments). The TER value reached a plateau when TJ barrier integrity was well established among cells, indicating maximal values and readiness for further experiments.

### Paracellular permeability assay

For the in vitro permeability assay, FD4, FD40 or RD70 (1 g/L) was added into the lower chamber once a confluent monolayer was formed, and incubated for 2 h. The apical solution was collected, and fluorescent intensity was determined using an EnSpire Multilabel Plate Reader (PerkinElmer). The apparent permeability coefficient (Papp) was calculated based on the increase in tracer amount per unit time and per filter area.^10^ To visualize the flux of macromolecules across monolayers, SMG-C6 cells were seeded onto 6-well plates or confocal dishes pre-coated with biotinylated-gelatin.^55^ For the cells seeds in 6-well plates, avidin-FITC (final concentration, 25 µg/mL) was added to the medium and incubated for 3 and 6 min. The tracer solution was removed, and the wells were washed with PBS before fixed with 4% paraformaldehyde for 15 min. The cells were then incubated with Alexa Fluor 594 conjugated claudin-4 antibody for 2 h at room temperature. For the cells seeded in confocal dishes, the nucleus and cell membrane were stained before the addition of avidin-FITC. The flux of avidin-FITC was observed in real-time in living cells using a confocal microscope (Leica Stellaris 8).

For the in vivo permeability assay, mice were treated with lipopolysaccharide (LPS, 5 mg/kg body weight) for 30 min. RD40 (0.5 mg/g body weight) was then injected into the angular vein. The lateral SMG was separated and placed in a glass chamber under a 2-photon laser-scanning microscope (Leica TCS-SP8 DIVE) following the previously described protocol.^56^

### Oligonucleotide transfection

MiR-145 mimics, inhibitors, and negative control oligonucleotides (each at 20 μmol/L) were designed and synthesized by RiboBio. Oligonucleotide transfection was performed by using riboFECT CP Transfection Kit (RiboBio). SMG-C6 cells were seeded in 12-well plates and incubated until they reached 40%-80% confluence before transfection. First, the transfection complexes were prepared and incubated at room temperature for 10 min. Then, the transfection complexes were slowly added to complete medium (without double antibodies) and subsequently added to each well containing cells. After 24 h of transfection, SMG-C6 cells were stimulated with IFN-γ for 24 h for subsequent experiments.

### Dual-luciferase reporter assay

Wide type (WT) and mutant (MUT) tricellulim-3’ UTR sequences were designed, synthesized, and inserted into the luciferase reporter vector pmirGLO (HanBio). 293 T cells was co-transfected with a mixture of firefly and Renilla luciferase reporters and the miR-145 mimic. Cells were incubated for 24 h, and the relative luciferase activity was determined using a dual-luciferase reporter assay kit (Promega) according to the manufacturer’s protocols. Firefly luciferase activity was first detected by adding Luciferase Assay Reagent II to the sample, producing a light signal that lasted for at least 1 min. After quantifying the fluorescence intensity of firefly luciferase, the reaction was terminated by adding Stop & Glo® Reagent to the same sample, initiating the Renilla luciferase reaction. The fluorescence intensity of the same sample was then quantified again.

### Statistical analysis

Data are presented as means ± SEM or medians using GraphPad Prism software. Normality was tested before analysis. Student’s *t* test, one- or two-way ANOVA followed by Dunnett’s, Turker’s or Holm-Šídák’s test for multiple comparisons were used, where *P* < 0.05 was considered significant. Mann-Whitney’s or Kruskal-Wallis’ tests were used for nonparametric data.

## Supplementary information

The transport of avidin-FITC (green) in rat submandibular gland epithelial polarized cell line SMG-C6

Supplemental materials
