# Supplementary material for: Loss of tricellular tight junction tricellulin leads to hyposalivation in Sjögren’s syndrome
Source: Int J Oral Sci. 2025 Mar 19;17:22. doi: 10.1038/s41368-025-00349-9 (PMC11923234; doi:10.1038/s41368-025-00349-9)
Supplement: Supplementary file 2 — Supplemental materials [file 41368_2025_349_MOESM2_ESM.pdf]

Loss of tricellular tight junction tricellulin leads to hyposalivation in Sjögren's syndrome

Xiang-Di Mao[1], Hai-Bing Li[1], Sai-Nan Min[2], Jia-Zeng Su[2], Pan Wei[3], Yan Zhang[1], Qi-Hua He[4], Li-Ling Wu[1], Guang-Yan Yu[2], Xin Cong[1 2*]

[1]Department of Physiology and Pathophysiology, Peking University School of Basic Medical Sciences, State Key Laboratory of Vascular Homeostasis and Remodeling, Beijing, 100191, P.R. China

[2]Department of Oral and Maxillofacial Surgery, Peking University School and Hospital of Stomatology & National Center of Stomatology & National Clinical Research Center for Oral Diseases & National Engineering Research Center of Oral Biomaterials and Digital Medical Devices, Beijing, 100081, P.R. China

[3]Department of Oral Medicine, Peking University School and Hospital of Stomatology & National Center of Stomatology & National Clinical Research Center for Oral Diseases & National Engineering Research Center of Oral Biomaterials and Digital Medical Devices, Beijing, 100081, P.R. China

[4]State Key Laboratory of Natural and Biomimetic Drugs, Peking University, Beijing, 100191, P.R. China

*Corresponding Author:

Xin Cong, Department of Physiology and Pathophysiology, Peking University School of Basic Medical Sciences, Beijing, 100191, P. R. China. Phone: 86-010-82802403, Fax: 86-010-82802403.

Email: congxin@bjmu.edu.cn

Supplemental

Supplemental Figures

Supplemental Fig. 1

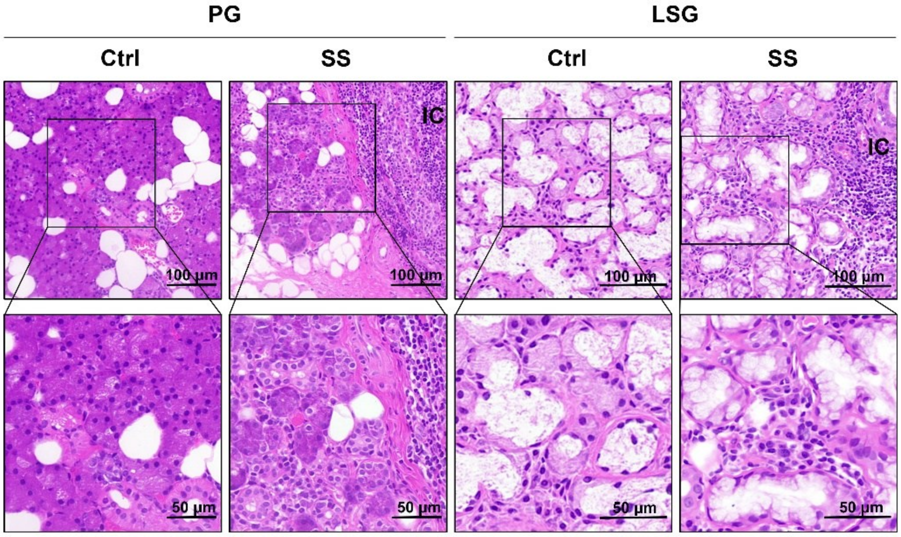

Supplemental Fig. 1 Hematoxylin and eosin (H&E) staining of labial salivary glands (LSGs) and parotid glands (PGs) from Sjögren's syndrome (SS) patients. The enlarged images (bar: 50 μm) were derived from boxes in the upper panels (bar: 100 μm). Ctrl, control. IC, infiltrating cells.

Supplemental Fig. 2

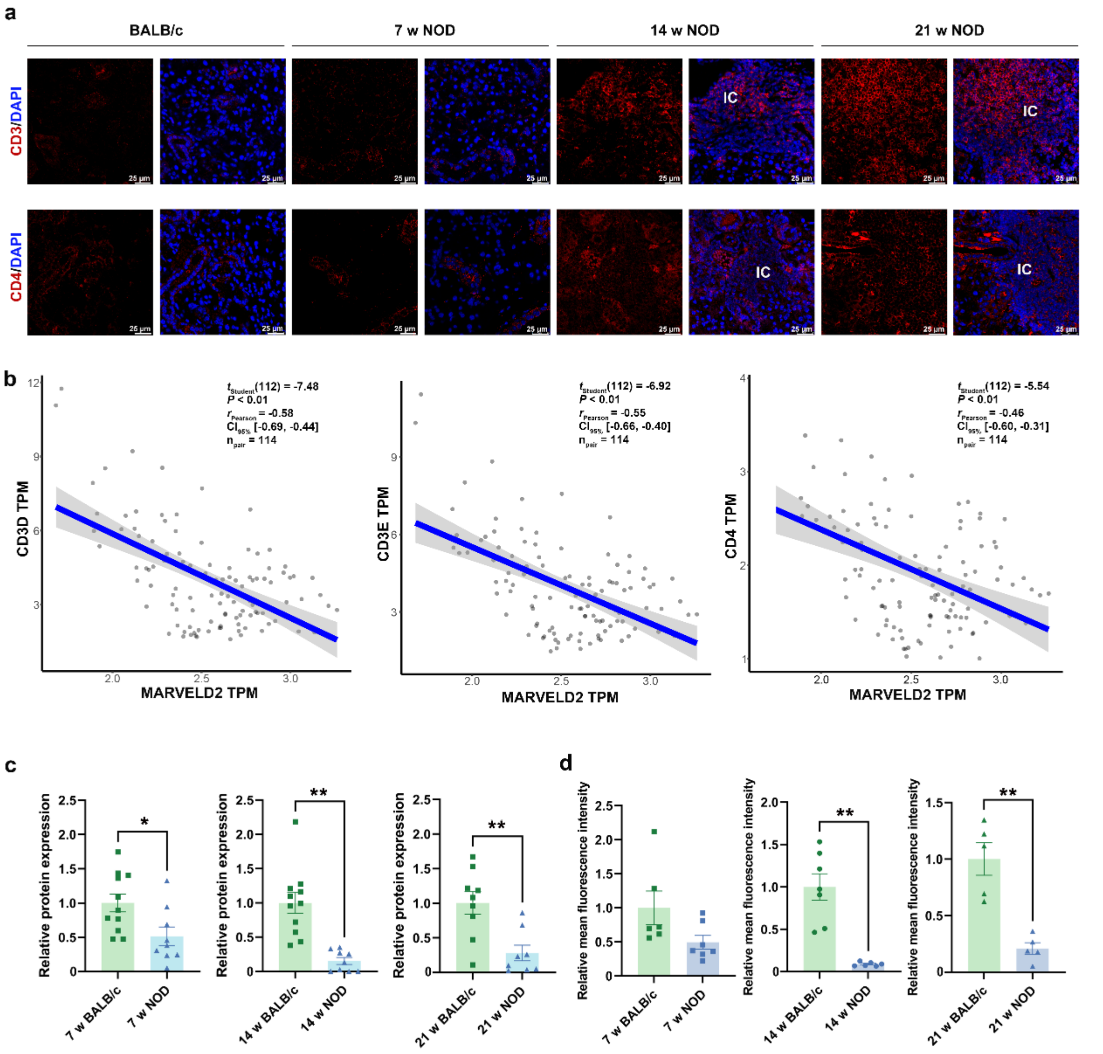

Supplemental Fig. 2 The expression of tricellulin in BALB/c and non-obese diabetic (NOD) mice with different ages. (**a**) The immunostaining of CD3 and CD4 for T cells and $CD4^+$ T cells (Th) cells. IC, infiltrating cells. (**b**) The correlation analysis between tricellulin gene expression and T cell infiltration according to public datasets (GSE173808 and GSE208260). CD3D and CD3E are two subunits of CD3. TPM, Transcript per million. (**c**) The grey intensity of tricellulin measured by the Image J software according to **Fig. 2g**. n=8-11. (**d**) The relative quantification of tricellulin measured by the Image J software according to **Fig. 2i** (bar: 25 μm). n=5-7. Analysis was performed unpaired two-tailed t test (**c-d**) where $*P < 0.05$ and $**P < 0.01$. The data are presented as means ± SEM (**c-d**).

Supplemental Fig. 3

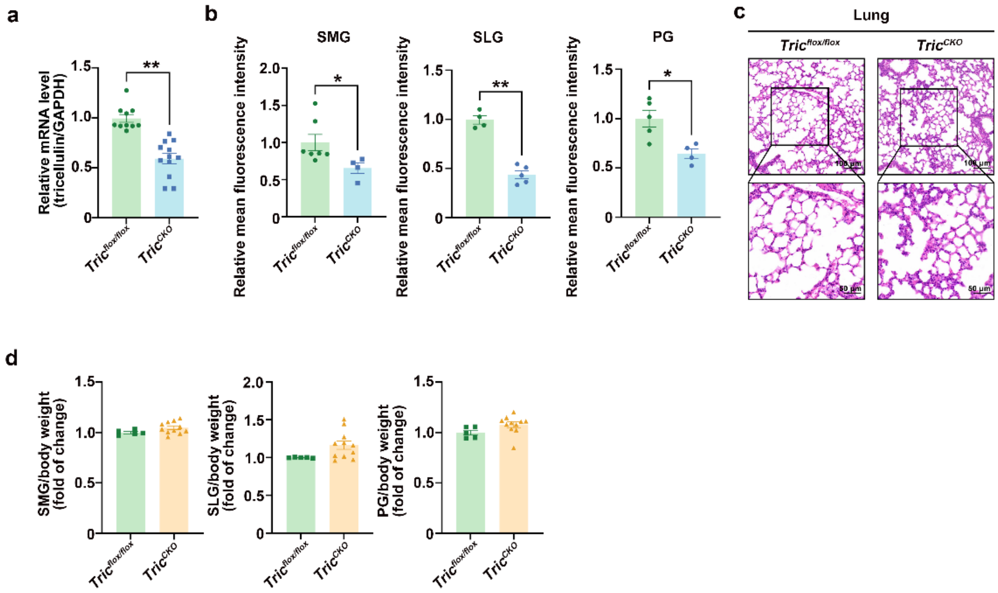

Supplemental Fig. 3 The manifestations of tricellulin in lungs and gross features of salivary glands of salivary gland acinar cell-specific tricellulin conditional knockout (*Tric*^{CKO}) mice. (**a**) The mRNA level of tricellulin in lung. n=9-10. (**b**) The semiquantitative analysis of tricellulin fluorescent intensity in salivary glands according to **Fig. 3d** (bar: 25 μm). (**c**) Hematoxylin and eosin (H&E) staining of lungs. (**d**) The ratio of salivary gland weight to body weight in mice. n=5-11. Analysis was performed by using two-tailed *t* test (**a-b, d**) where *$P < 0.05$ and **$P < 0.01$. The data are presented as means ± SEM. SMG, submandibular gland. SLG, sublingual gland. PG, parotid gland.

Supplemental Fig. 4

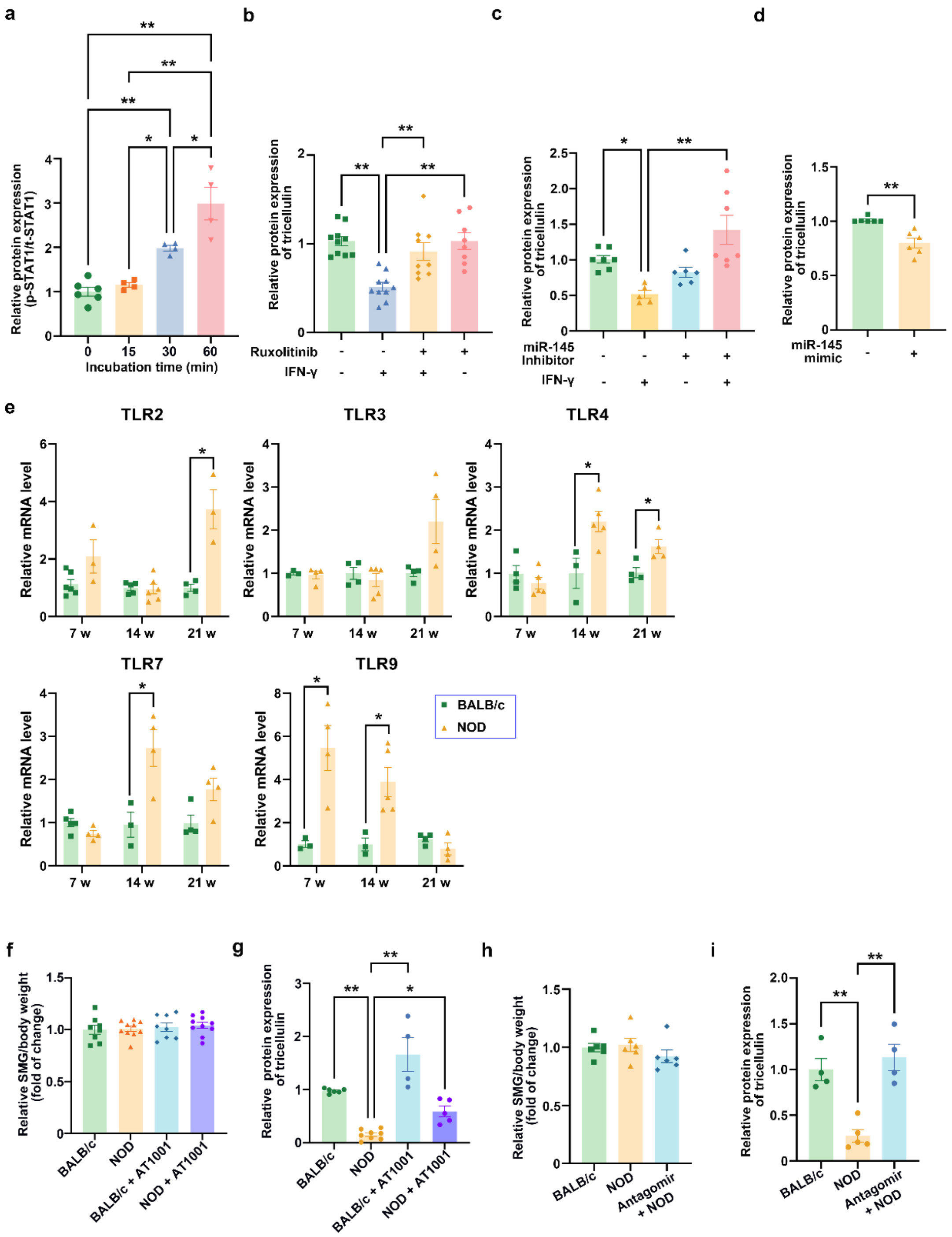

The regulatory mechanism of interferon-γ (IFN-γ) on tricellulin in salivary glands. (**a**) The grey intensity of phosphorated signal transducer and activator of transcription (p-STAT1) measured by the Image J software according to

Fig. 7a. n=4-6. (**b**)The grey intensity of tricellulin measured by the Image J software according to **Fig. 7b**. n=8-10. (**c**, **d**) The grey intensity of tricellulin regulated by miR-145 inhibitor (**i**) and mimic (**j**) measured by the Image J software according to **Fig. 7i** and **Fig. 7j** respectively. n=5-7. (**e**) The mRNA levels of Toll-like receptors (TLRs) in NOD mice. n=4-5. (**f**) The ratio of submandibular gland (SMG) weight to body weight in mice. n=8-10. The intraperitoneal injection of tight junction sealer AT1001 was administrated into 8-week-old NOD mice once a day for two weeks. (**g**) The grey intensity of tricellulin measured by the Image J software according to **Fig. 8f**. n=4-8. (**h**) The ratio of SMG weight to body weight in mice. The intraperitoneal injection of miR-145 antagomir was administrated into 6-week-old NOD mice twice a week for one month. n=4-6. (**i**) The grey intensity of tricellulin measured by the Image J software according to **Fig. 8n.** Analysis was performed by using one-way ANOVA (**a-c, e, f, h-i**), using two-tailed *t* test (**d, e**) and where $*P < 0.05$ and $**P < 0.01$. The data are presented as means ± SEM. Tric, tricellulin. NOD, non-obese diabetic.

Supplemental Tables

Supplemental Table 1. Clinical characteristics of patients with Sjögren's syndrome

Number	Gender	Numbers of lymphofoid focus	Biopsy	Diagnosis
1	Female	1	LSG	Sjögren's syndrome
2	Female	2	LSG	Sjögren's syndrome
3	Female	2	LSG	Sjögren's syndrome
4	Female	2	LSG	Sjögren's syndrome
5	Female	2	LSG	Sjögren's syndrome
6	Female	>2	LSG	Sjögren's syndrome
7	Female	>2	PG	Sjögren's syndrome

Abbreviations: LSG, labial salivary gland. PG, parotid gland.

Supplemental Table 2. The primers used for qPCR

Species	Gene	Forward primer (5’-3’)	Reverse primer (5’-3’)
Mus musculus	Tric	CAGGAACTCCCGATGTCT	TCTGCTACTACCCGTTATTT
	Ocln	CCATCTTTCTTCGGGTTTTCA	CTTCTGGATCTATGTACGGCTCA
	ZO-1	GTAAAGCCTGGTGGTGGAACT	TCGAACCTCTACTCTACGACATG
	Cldn1	GACAGGAGCAGGAAAGTAGGA	CTTTGGAATTAGGCAGAACGA
	Cldn3	TTTCTTTGTCCATTCGGCTTG	ACCGTACCGTCACCACTACCA
	Cldn4	GATCTTGGCCTTGACGGTCTC	CTCTGGATGAACTGCGTGGTG
	F11r	TCTCTTCACGTCTATGATCCTGG	TTTGATGGACTCGTTCTCGGG
	Lsr	AGTGACACTACACTGCACCTA	CCCGACAGAACGACTTATACTTC
	Ildr1	GTTTGCCTCCGTTACCCTCA	GGGACAAAGCTGCCTGGTAT
	Ildr2	CAGCCCACTGTGCTTCGAT	AGGATTTGAACTTCCACTGAACC
	IFN-γ	ACTGGCAAAAGGATGGTG	GTTGCTGATGGCCTGATT
	TNF-α	AGCCCCCAGTCTGTATCCTT	CTCCCTTTGCAGAACTCAGG
	IL-1β	GCCCATCCTCTGTGACTCAT	AGGCCACAGGTATTTTGTCG
	IL-6	AGCCCACCAAGAACGATAG	GGTTGTCACCAGCATCAGT
	TLR2	CACTGGGGGTAACATCGCTT	AGTCAGGTGATGGATGTCGC
	TLR3	ACCTCCAGAAGAAC	GAACGGATTGAAGCGCAT

		CTCATAAC	ATC
	TLR4	TGGCTGGTTTACACGTCCAT	TGCAGAAACATTCGCCAAGC
	TLR7	TGTGCCCCCAACATGGTTTA	ACATACCCCTTGACACGCAG
	TLR9	GCGCCCAAACTCTCCCTTAT	CCTCCAGACACAAGCGTAGG
Rat	Tric	ACTCTGCTACTACCCGTTA	ACACATTCTCCTTTTTGAC
	Ocln	ACCTTGTCCGTGGATGACTTC	TAGTCTCCCACCATCCTCTTG
	ZO-1	GTATCCGATTGTTGTGTTCCTT	AGACCGTTCATATAGCTTCCTG
	Cldn1	CTGGGTTTCATCCTGGCTTCGC	CACTGTATCTGCCCGGTGCTTT
	Cldn3	GCCCAGTGTACCAACTGCGTAC	ACCAGCGGGTTATAAAATCCC
	Cldn4	TGCTGATTATGGTGCCTGTGTC	CGGAGTAGGGCTTTTCGTTGCG
	F11r	CTCCTCTGTCACCATTGGG	ACGGGTTTCTTGGCATCT
	Lsr	CAACAGCAATGAGCCACACC	CCCCACCTACATCCCTGGTA
	Ildr1	CTCTGAGGTCGTGGAACGC	CGATGGGTCCAACTCCCTTC
	Ildr2	ATGTGAGATTTGGGCAGGGA	GGACCTCACCTTCCATCACA
	GAPDH	CTTTGGCATTGTGGAAGGGCTC	GCAGGGATGATGTTCTGGGCAG

Abbreviations: Tric, tricellulin. Ocln, occludin. Tjp1, tight junction protein 1. Cldn, claudin. F11r, junctional adhesion molecule 1. Lsr, lipolysis stimulated lipoprotein receptor. Ildr, immunoglobulin like domain containing receptor. IFN-γ, interferon-γ. TNF-α, tumor necrosis factor-α. IL-6, interleukin-6. IL-1β, interleukin-1β. TLR, Toll-

like receptor.
